# Examinations by Teachers

**Published:** 1905-06-03

**Authors:** 


### EXAMINATIONS BY TEACHERS.

The Education Committee of the General Medical Council has presented to that body a report on "Teaching without Examination" which incidentally opens out if not a likelihood, at least a possibility, of a radical change in the time-honoured English methods of ascertaining the fitness of candidates to receive qualifications entitling them to practise medicine. The Committee was requested to inquire into the possibility of including certain subjects in the curriculum without requiring examination in them. On this point they have little to say beyond the statement that certain subjects—namely, 'Vaccination, Ophthalmology, Mental Diseases (the Committee presumably mean insanity), and Infectious Diseases, although required as parts of the curriculum, are not specially examined upon, although questions relating to them may be put; but they go on to say that, in civilised countries generally, "the examination is never divorced from the teaching, and the record of the student's work in the schoolroom, laboratory, and hospital is always a most important factor in the ultimate decision" concerning him. They also say, on the authority of Dr. H. Frank Heath, of the Education Department, that the only real parallel to the methods pursued in the United Kingdom is the examination system of China; and they bring out the fact that the examination of students abroad is almost entirely—generally entirely—in the hands of those who have taught them. It was thereupon moved by Dr. Mackay, and seconded by Sir John Tuke, that a committee should be asked to prepare a detailed report upon the system of examination in medicine followed in other countries, with the special object of determining the number of professional examinations demanded, the subjects included in those examinations, and the value (if any) attached to the records of class attendance and work. The motion was lost; but it is idle to suppose that a question of such importance can be shelved indefinitely. It goes to the very root of medical efficiency.

There is probably not a single medical teacher in the United Kingdom who could not recall examples of men who were successful at the examinations now in force but who, in his estimation, were totally unfit to be entrusted with the responsibilities of the profession, or who could not recall examples of an opposite character—that is to say, of men who had failed when they deserved to succeed. We all know that there is only one method of acquiring trustworthy knowledge, and that method is by dint of steady work. Any amount of information otherwise obtained is but a simulacrum of knowledge, and will inevitably break down under trial. Now the weak point of the British (or Chinese) system is that it affords no evidence of work—no certain means of distinguishing between ingrain and varnish. The field of medical science is so much larger than the possible scope of any single examination that a badly prepared candidate trusts to his luck to escape from questions concerning which he may be conscious of ignorance. Taking the subjects mentioned by the Committee, it is obvious that a student may have been a stranger to the ophthalmic department of his hospital, and that he may nevertheless pass an examination in which no single question relating to ophthalmology has been included, and may go into practice and treat eye-disease on the strength of it. In an examination by teachers it is equally obvious that a knowledge of the candidate's shortcomings would lead to endeavours to probe his weaknesses as well as to display his strength, and it would also have the incidental effect of calling his special attention, during the course of study, to departments of work which he was apparently neglecting. It may probably be said of the present system that nearly every student, however incapable, passes the examination of some licensing body at last. The examiners know nothing of his conduct, they get tired of seeing him, and he learns in time to escape pitfalls which may be fatal to inexperience. It would be an excellent thing to have in medicine, as in some other callings, a time-limit, fixed either by age or by length of so-called study, or by number of failures, after the lapse of which the candidate should be finally and permanently excluded. Failing this, we are disposed to think that systematic examination by teachers would be an immense improvement upon our present method of knowledge-testing; and the fact that it appears to obtain in every other part of the civilised world may, surely, be regarded as affording valuable testimony to its advantages.

